# Universal Coverage without Universal Access: Institutional Barriers to Health Care among Women Sex Workers in Vancouver, Canada

**DOI:** 10.1371/journal.pone.0155828

**Published:** 2016-05-16

**Authors:** M. Eugenia Socías, Jean Shoveller, Chili Bean, Paul Nguyen, Julio Montaner, Kate Shannon

**Affiliations:** 1 British Columbia Centre for Excellence in HIV/AIDS, St. Paul’s Hospital, Vancouver, BC, Canada; 2 Department of Medicine, University of British Columbia, St. Paul’s Hospital, Vancouver, BC, Canada; 3 School of Population and Public Health, University of British Columbia, Vancouver, BC, Canada; 4 Sex Workers United Against Violence Society, Vancouver, BC, Canada; UNAIDS, TRINIDAD AND TOBAGO

## Abstract

**Background:**

Access to health care is a crucial determinant of health. Yet, even within settings that purport to provide universal health coverage (UHC), sex workers’ experiences reveal systematic, institutionally ingrained barriers to appropriate quality health care. The aim of this study was to assess prevalence and correlates of institutional barriers to care among sex workers in a setting with UHC.

**Methods:**

Data was drawn from an ongoing community-based, prospective cohort of women sex workers in Vancouver, Canada (An Evaluation of Sex Workers’ Health Access). Multivariable logistic regression analyses, using generalized estimating equations (GEE), were employed to longitudinally investigate correlates of institutional barriers to care over a 44-month follow-up period (January 2010-August 2013).

**Results:**

In total, 723 sex workers were included, contributing to 2506 observations. Over the study period, 509 (70.4%) women reported one or more institutional barriers to care. The most commonly reported institutional barriers to care were long wait times (54.6%), limited hours of operation (36.5%), and perceived disrespect by health care providers (26.1%). In multivariable GEE analyses, recent partner- (adjusted odds ratio [AOR] = 1.46, % 95% Confidence Interval [CI] 1.10–1.94), workplace- (AOR = 1.31, 95% CI 1.05–1.63), and community-level violence (AOR = 1.41, 95% CI 1.04–1.92), as well as other markers of vulnerability, such as self-identification as a gender/sexual minority (AOR = 1.32, 95% CI 1.03–1.69), a mental illness diagnosis (AOR = 1.66, 95% CI 1.34–2.06), and lack of provincial health insurance card (AOR = 3.47, 95% CI 1.59–7.57) emerged as independent correlates of institutional barriers to health services.

**Discussion:**

Despite Canada’s UHC, women sex workers in Vancouver face high prevalence of institutional barriers to care, with highest burden among most marginalized women. These findings underscore the need to explore new models of care, alongside broader policy changes to fulfill sex workers’ health and human rights.

## Introduction

As a basic human right and a critical determinant of individual and population health outcomes [1], Universal Health Coverage (UHC) is the subject of a globally approved United Nations General Assembly resolution (A.67/81) [2], and has emerged as a key component of the 2030 Sustainable Development Goals [3]. Accumulating evidence suggest that access to health services is a multidimensional concept that results from the interaction between individual factors, social and physical living and working environments, the characteristics of the health system, and macro-structural-level factors (e.g., laws and policies) [4–6]. Among these, health system-related factors, including service accessibility (e.g., distance/transportation), availability (e.g., waiting times), and acceptability (e.g., language and cultural barriers, enacted and perceived stigma) have been acknowledged as particularly important from a health policy perspective as they concomitantly disenfranchise those most in need, while being most amenable to institutional-level interventions (e.g., funding formulae; hospital policies; legal frameworks) [7, 8].

Although Canada is frequently described as a leader in the realization of UHC [9], in reality, many Canadians, including women, recent immigrants, Aboriginal people, and youth, face multiple institutionally-generated barriers when trying to access good quality and appropriate health services [10–15]. As a result, many postpone or forgo seeking care, with potentially catastrophic impacts on their health [16, 17].

Research shows that in Canada, and in other contexts that criminalize sex work, sex workers bear an array of health and social harms, including violence, exposure to HIV and other STIs, and substance use that require attention within the health care system [18–21]. While access to health care has been identified as a key determinant of sex workers’ health [18, 22], research on institutional-level barriers that affect sex workers’ access to appropriate and high-quality health care remains limited, particularly in settings with UHC. To address this gap, the current study documents the prevalence and correlates of institutional-level barriers to health services among a prospective cohort of street- and off-street sex workers in Vancouver, Canada.

## Materials and Methods

### Study design, population and procedures

An Evaluation of Sex Workers’ Health Access (AESHA) is an ongoing community-based, prospective cohort of sex workers beginning in 2010, that was developed based on community collaborations with sex work agencies since 2005, and that has been described in detail previously [23]. In brief, using time-location sampling [24, 25], sex workers are recruited through outreach during alternate working hours to outdoor/public (e.g., streets, alleys), indoor (e.g., massage parlours, micro-brothels), and off-street (e.g., online and newspapers advertisements) sex work venues across Metro Vancouver. These venues are identified and regularly updated through community mapping with current/former sex workers. Individuals aged 14 years and older, who self-identify as women, have exchanged sex for money in the previous 30 days at baseline, and provide written informed consent, are eligible for inclusion.

After providing written informed consent, at baseline, and on a bi-annual basis thereafter, participants complete an interviewer-administered questionnaire, followed by voluntary HIV/HCV/STI testing. Basic treatment for STIs is also offered onsite, regardless of participation in the study. Interview, outreach and nursing staff include both experiential (current/former sex workers) and non-experiential staff with substantial community rapport. The questionnaire collects socio-demographic data, sex work and drug use patterns, and physical, social-structural characteristics of the working and living environment, as well as information on overall health and wellness, and health services access and utilization. Participants receive an honorarium of $40CAD for their time and expertise at each study visit. The study has been approved by the Providence Health Care/University of British Columbia Research Ethics Board, and is monitored by a community advisory board of 15+ women, sex work and policy partner agencies. For the current analysis, cohort participants who completed at least one study visit between January 1, 2010 and August 31, 2013 were eligible for inclusion. Thus, the cohort data used in this analysis was collected during the same time frame.

### Study variables

The primary outcome of interest for this study was a time-updated variable (using the prior 6 months as a reference point) of having experienced one or more institutional-level barriers to accessing health care. Institutional-level barriers were categorized in two broad ways: (1) Availability of care (operationalized as having experienced no or poor access to care due to any or all of the following: limited hours of operation or limited number of physicians at clinical site(s), and long wait times); and (2) Acceptability of care (operationalized as having experienced poor quality care due to any or all of the following: was not served in preferred language, health care provider of preferred sex/gender was not available, and felt disrespected by health care providers).

Based on prior literature examining access to health care we considered a number of independent variables that have been shown to influence access to health services, with a particular focus on social-structural-level factors [4, 5, 22]. Time-fixed variables of interest at baseline included: socio-demographic characteristics, such as: age, Indigenous/Aboriginal ancestry; sexual/gender identity (lesbian, gay, bisexual, transgender* or two-spirit,—LGBT*2S—versus cis-gender and straight); and migration status (immigrant versus Canadian-born). All other variables considered were time-updated variables at each semi-annual follow-up using the last 6 months as a reference point, and were dichotomized (yes versus no) unless otherwise specified. These include: individual medical comorbidities (HIV and HCV sero-status, and self-reported lifetime diagnosis of mental illness, including depression, post-traumatic stress disorder, anxiety, schizophrenia, and borderline personality); individual behaviours, such as non-injection or injection drug use; and interpersonal-level risks, such as physical/sexual violence by partners or clients. We also accounted for other previous experiences of violence, including having been threatened/verbally assaulted by community residents or businesses, self-reported police harassment, arrest, and incarceration. Furthermore, the analysis included other structural-level factors, such as unstable housing (i.e., ≥1 night in a single room occupancy hotel, shelter, treatment/recovery house, couch surfing, staying in a vehicle, on the street/alley/park), financially supporting dependents (e.g., child or partner), having a provincial health insurance card; as well as physical and social features of the environments where sex workers provide primarily services (formal sex work establishment, such as massage/beauty parlours or micro-brothels; informal indoor venues, such as sauna, bar/clubs, hotel/hourly rental, or clients’ house; or outdoor/public space, such as street, public washroom, or car).

### Statistical Analyses

As a first step we examined individual-, interpersonal- and structural-level factors among participants at baseline, stratified by having experienced institutional-level barriers to care at some point during the study period. Since analyses of factors potentially associated with reporting institutional barriers to health care included serial measures for each participant, we then ran bivariate and multivariable logistic regression using generalized estimating equations (GEE) analyses with a logit link for the dichotomous outcome. The GEE method provides standard errors adjusted for the repeated measurements from the same participant using an exchangeable correlation structure. Variables found to be associated with institutional-level barriers at p <0.10 level in bivariate analyses were considered for inclusion into the multivariable model. As in previous research [26, 27], the multivariable model was built using a backward selection approach. Quasi-likelihood under the independence model criterion (QIC) was used to identify the model with the best overall fit as indicated by the lowest QIC value [28]. A complete case analysis approach was employed, where cases with missing observations were excluded from the multivariable analyses. This reduced the data set available for the multivariable analysis from 2506 to 2457 observations. All statistical analyses were performed using the SAS software version 9.4 (SAS Institute, Cary, NC, USA).

## Results

**Fig 1:**
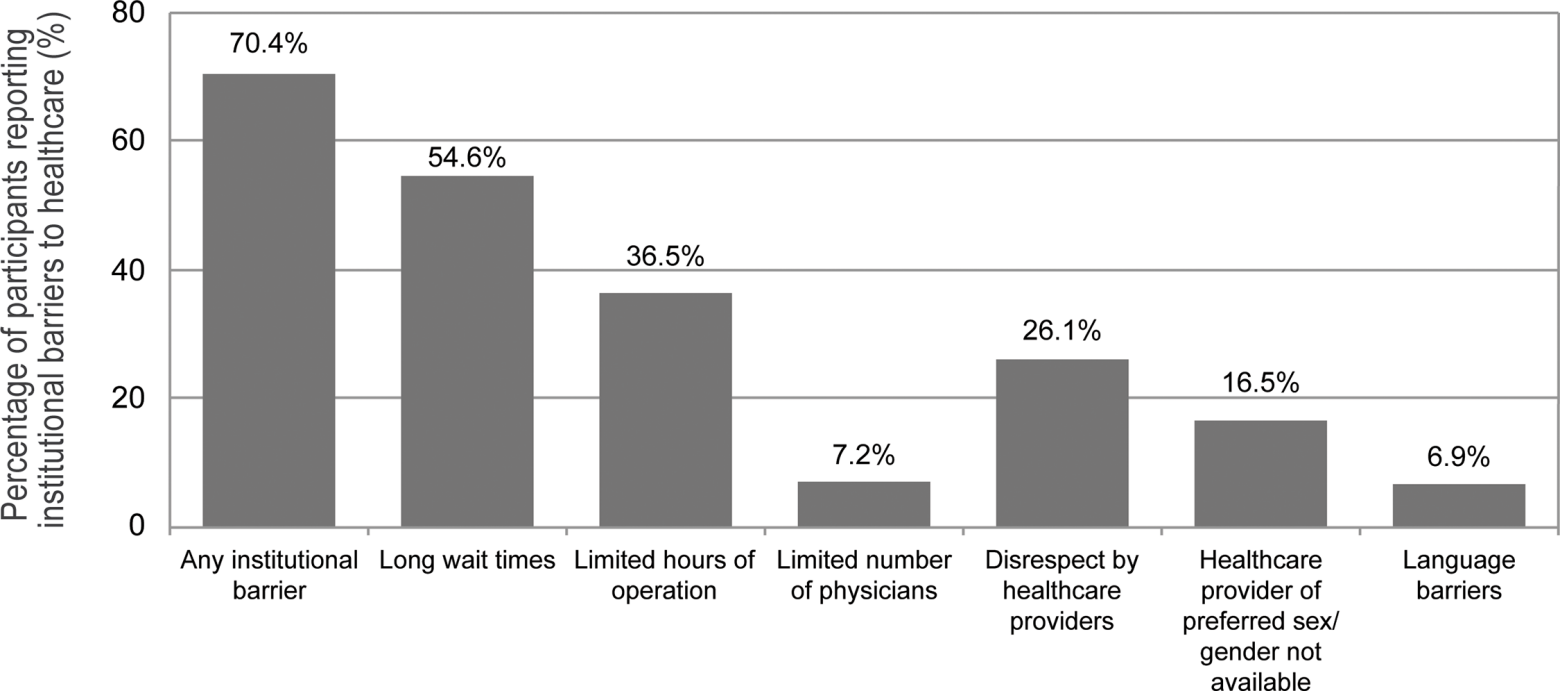
Frequency of institutional-level barriers to health care among sex workers in Vancouver, Canada, 2010–2013.

In total, 723 sex workers were included, with a median age of 34.5 years (Interquartile range [IQR] 28.0–42.0). As shown in Table 1, over a third were of Indigenous ancestry (35.8%), and over a quarter were immigrants to Canada (27.4%). Overall, 11.2% participants were living with HIV and 41.8% with HCV. Recent use of non-injection and injection drugs at the time of enrolment was relatively high, 68.6% and 39.4% respectively. These 723 sex workers contributed to 2506 observations for this analysis. The median duration of follow-up per participant was 18.2 months (IQR 0–30.8) months, which corresponds to a median number of study visits of 3 (IQR 1–5). Over the study period, there were 1097 institutional-level barrier events (43.8%), with 509 participants (70.4%) reporting at least one time when they could not access care due to institutional-level barriers. As indicated in Fig 1, the most frequently reported institutional-level barriers were those related to service availability including long wait times (54.6%) and limited hours of operation (36.5%). In addition, over one-quarter of study participants reported having been unable to access acceptable health care due to feeling disrespected by health care providers (26.1%).

**Table 1:** Baseline characteristics of sex workers, stratified by self-reported institutional-level barriers to health care at some point during the study period, Vancouver, Canada, 2010–2013.

Characteristic	Total, n (%)† (N = 723)	Institutional barriers to health care*, n (%)	
		Yes (n = 509)	No (n = 214)
***Individual-level factors***			
Age <25 years old	96 (13.3)	63 (12.4)	33 (15.4)
Indigenous ancestry	259 (35.8)	196 (38.5)	63 (29.4)
Sexual/gender minority	184 (25.5)	146 (28.7)	38 (17.8)
Immigrant to Canada	198 (27.4)	114 (22.4)	84 (39.3)
HIV positive*	81 (11.2)	56 (11.0)	25 (11.7)
HCV positive*	302 (41.8)	232 (45.6)	70 (32.7)
Mental health illness*	347 (48.0)	282 (55.4)	65 (30.4)
Non-injection drug use*	496 (68.6)	376 (73.9)	120 (56.1)
Injection drug use*	285 (39.4)	227 (44.6)	58 (27.1)
***Interpersonal-level factors***			
Physical/sexual violence by partners*	109 (15.1)	89 (17.5)	20 (9.4)
Physical/sexual violence by clients*	168 (23.2)	131 (25.7)	37 (17.3)
***Structural-level factors***			
Unstable housing*	590 (81.6)	420 (82.5)	170 (79.4)
Supports others financially*	209 (28.9)	133 (26.1)	76 (35.5)
No provincial health insurance card*	12 (1.7)	7 (1.4)	5 (2.4)
Primary place of servicing clients*			
Formal sex work/in-call establishment	222 (30.7)	127 (25.0)	95 (44.4)
Informal indoor venue	189 (26.1)	141 (27.7)	48 (22.4)
Outdoor/public space	312 (43.2)	241 (47.4)	71 (33.2)
Threatened/ verbally assaulted by community residents or businesses*	103 (14.3)	90 (17.7)	13 (6.1)
Police harassment without arrest*	272 (37.6)	204 (40.1)	68 (31.8)
Police arrest*	49 (6.8)	40 (7.9)	9 (4.2)
Incarceration*	108 (14.9)	82 (16.1)	26 (12.2)

* Time-updated variable using last 6 months as a reference point

† Percentages may not necessarily sum to 100% due to missing observations or rounding error.

Table 2 presents results of the bivariate and multivariable GEE logistic regression analyses. In the bivariate analysis, factors positively associated with experiencing institutional barriers over the study period included self-identification as a gender/sexual minority, having ever been diagnosed with a mental illness, recent use of injection drugs, having experienced violence by intimate partners and/or clients, not having a provincial health insurance card, having been threatened by community residents or businesses, and having been harassed or arrested by the police. On the contrary, women living with HIV and immigrant participants had reduced odds of experiencing these barriers.

**Table 2:** Bivariate and multivariable GEE logistic regression analyses of correlates of self-reporting institutional barriers to health care among a prospective community cohort of sex workers in Vancouver, Canada, 2010–2013.

Characteristic	Odds Ratio (95% CI)	
	Unadjusted	Adjusted‡
***Individual-level factors***		
Age <25 years old (yes vs. no) †	1.35 (0.96–1.88)	
Indigenous ancestry (yes vs. no)	0.96 (0.78–1.20)	
Sexual/gender minority (yes vs. no) †	1.49 (1.17–1.89)	1.32 (1.03–1.69)
Immigrant to Canada (yes vs. no) †	0.78 (0.61–1.00)	
HIV-positive (yes vs. no) *†	0.57 (0.42–0.78)	0.54 (0.39–0.72)
HCV-positive (yes vs. no) *	0.86 (0.69–1.06)	
Mental health illness (yes vs. no) *†	1.79 (1.45–2.20)	1.66 (1.34–2.06)
Non-injection drug use (yes vs. no) *	1.07 (0.86–1.32)	
Injection drug use (yes vs. no) *†	1.22 (1.01–1.48)	
***Interpersonal-level factors***		
Physical/sexual violence by partners (yes vs. no) *†	1.70 (1.30–2.21)	1.46 (1.10–1.94)
Physical/sexual violence by clients (yes vs. no) *†	1.53 (1.25–1.88)	1.31 (1.05–1.63)
***Structural-level factors***		
Unstable housing (yes vs. no) *	1.15 (0.95–1.40)	
Supports other financially (yes vs. no) *	1.13 (0.93–1.38)	
No provincial health insurance card (yes vs. no) *†	2.19 (1.02–4.72)	3.47 (1.59–7.57)
Primary place of servicing clients (ref: formal sex work establishment/in-call venue) *		
informal indoor venue	1.07 (0.83–1.37)	
outdoor/public space	1.20 (0.93–1.57)	
Threatened/ verbally assaulted by community residents or businesses (yes vs. no) *†	1.73 (1.30–2.30)	1.41 (1.04–1.92)
Police harassment without arrest (yes vs. no) *†	1.30 (1.09–1.54)	
Police arrest (yes vs. no) *†	1.89 (1.23–2.90)	1.52 (0.96–2.41)
Incarceration (yes vs. no) *	1.17 (0.91–1.49)	

* Time-updated variable using last 6 months as a reference point

† Significant at p <0.10 in the unadjusted analyses and considered as potential confounders in the multivariable model selection process.

‡ Only the final list of variables included in the multivariable model after variable selection is included in this column.

In the multivariable GEE model, self-identification as a gender/sexual minority (adjusted odds ratio [AOR] = 1.32, 95% Confidence Interval [CI] 1.03–1.69), having ever been diagnosed with a mental illness (AOR = 1.66, 95% CI 1.34–2.06), having experienced violence by intimate partners (AOR = 1.46, 95% CI 1.10–1.94) or clients (AOR = 1.31, 95% CI 1.05–1.63), not having a provincial health insurance card (AOR = 3.47, 95% CI 1.59–7.57), and having been threatened by community residents or businesses (AOR = 1.41, 95% CI 1.04–1.92) remained independently associated with increased odds of experiencing institutional barriers to care; while women living with HIV remained associated with reduced odds (AOR = 0.54, 95% CI 0.39–0.72).

## Discussion

Despite Canada’s universal health system, our results show that women sex workers in Vancouver face high prevalence of institutional barriers to health care. Over a 44-month follow-up period, seven of every ten participants reported institutional barriers to health services, approximately three-times higher than estimates of difficulty accessing care among the general Canadian population [11, 29]. Long wait times were the most frequently experienced institutional-level barrier, affecting more than half of the sex workers evaluated. Moreover, one quarter of study participants reported that feeling disrespected by health care providers interfered with their ability to access care. Alarmingly, those who may have the greatest and most complex health care needs, including sex workers with mental illnesses and victims of gender-based violence, were also more likely to report institutional barriers. Lack of training and sensitivity of health care providers to marginalized populations is concerning, and warrants immediate action at different levels, ranging from medical education and training to anti-discriminatory health policies.

A key and extremely relevant finding of the current study is that sex workers reporting having experienced violent events, either at the partner-, workplace- (i.e., client violence), or community-level (i.e., threatened by community residents or businesses), were at 30% to 50% increased odds of experiencing institutional barriers to health care. This is in line with previous research demonstrating links between partner- and workplace-based violence and reduced access to health services [20, 30–32]. These findings reflect the pervasiveness of violence, as well as ongoing stigma and discrimination against sex workers. Indeed, an emerging body of evidence indicates that sex workers suffer a disproportionate burden of violence compared to the general population of women, which is usually enhanced by the cultural taboos against the sell of sex and the criminalized or quasi-criminalized nature of sex work prevailing in many parts of the world [31, 33–35]. Fear of arrest or police harassment forces sex workers to work in more isolated and hidden spaces, limiting their ability to work together, and placing them at increased risk of violence [22]. Further, criminalization contributes to an environment, where violence against sex workers is seen as normal or justified [18]. Gender-based violence, in turn, is a well-known structural determinant of multiple adverse health outcomes, including increased risk for HIV infection, unintended pregnancies, mental health problems, and mortality [31, 36]. Collectively, these findings point to the importance of a human rights approach to the provision of health care, as well as other potential health-related benefits of decriminalizing sex work. Sex workers' safety and access to health services and other support resources is a public health imperative [19, 20, 37]. In addition, evidence from other settings suggests that community empowerment and other health-promoting institutional arrangements (e.g., sex work collectives and unions) may also buttress other macro-level reforms (e.g., legal reforms regarding sex work), as well as other efforts to address violence reduction and promote more accessible and acceptable forms of health care for sex workers [38–42]. Alongside these efforts to reduce violence, systems should be put in place to rapidly respond when an episode of violence occur, and facilitate access of victims of violence to non-judgemental health services, peer-based interventions, counselling and other relevant legal and social support [43].

Unsurprisingly, not having a provincial health insurance card was a strong correlate of reporting institutional-level barriers to health care. Despite Canada’s national publicly funded health system, a substantial number of individuals lack health insurance, mainly homeless and migrants with precarious status, all of whom might face multiple and intersecting health and social disparities [44–47]. Disrespectful treatment by health care providers, distance to available health services or limited knowledge on how to navigate them, as well as fear of being denounced to immigration among migrants with precarious status are obstacles frequently reported by these vulnerable groups [45, 46, 48, 49]. These barriers are often exacerbated among sex workers due to the criminalized and highly stigmatized nature of sex work in Canada [46, 47, 50]. In addition to being excluded from publicly funded coverage, uninsured individuals usually cannot seek private health insurance due to high costs and/or lack of citizenship status [45, 46, 48, 49]. Indeed, previous research in Vancouver shows that recent migrant women sex workers have 3-fold increased odds of unmet health care needs compared to Canadian-born counterparts [51]. While community-based health centres offer no-cost care, their long waiting lists and enrolment requirements (e.g., to live within clinic’s catchment area, to have identification documents) often result in postponement or avoidance of care [46]. Collectively, these data underscore the urgent need for structural-level interventions to remove barriers to care, particularly among medically uninsured sex workers. Models from a variety of settings, including the Mobile Access Project (MAP van) in Vancouver, Canada, the St. James Infirmary in San Francisco, CA, USA, and the Sonagachi Project in India, indicate that peer/ sex worker-driven models are critical to facilitate access to health services for hidden, stigmatized and highly mobile populations such as under housed and migrant sex workers [27, 52–55]. The elimination of waiting periods for provincial health insurance, alongside increases of human and financial resources allocated to community health clinics and relaxation of their enrolment criteria could go a long way to addressing the pressing health care needs of this vulnerable population [46].

In the current analysis, sexual/gender minority women were more likely to experience institutional-level barriers to care, reinforcing the urgent need for gender-sensitive and culturally appropriate care that is tailored to the special needs of sexual and gender minorities. Although, the LGBT community is not a homogenous group, they share a long history of pervasive marginalization and systematic exclusion, which continues to shape the numerous health inequities that affect this population [56–59]. LGBT populations face multiple challenges in accessing appropriate care, including refusal of care, harassment, and lack of competent and sensitive providers with adequate knowledge of their unique needs, which may further exacerbate these inequities [56, 57, 60–63]. Again, St. James Infirmary offers a best practices approach to sex worker-led occupational safety and health clinics for sex workers of all genders and sexual orientations [54]. Expanding and emphasizing LGBT-related topics in medical and nursing schools’ curricula, as well as recurrent training to health care workers could also contribute to a more knowledgeable and sensitive health care workforce. This, alongside the development of strong anti-discriminatory health policies will be critical for the achievement and sustainability of appropriate, safe and welcoming health service environments for all sex workers [64–66].

It is well known that untreated mental health needs can have significant negative health and socio-economic consequences, both for the individual and for the health system [67–71]. Thus, our findings that sex workers with mental disorders had increased odds of reporting institutional barriers to health services, including treatment for their mental health condition, is highly concerning. Uptake of mental health services are known to be affected by several factors, including those at the intra- and inter-personal level (e.g., stigma, distrust about the effectiveness of treatments, low self-perceived need for services), as well as institutional-level barriers (e.g., availability and accessibility of services) [67, 72, 73]. Importantly, institutional barriers seem to be more prevalent among individuals with severe/moderate illnesses [67, 74, 75]. In addition, while services by psychiatrists are covered under the Canada’s UHC, other mental health-related services, such as counselling or other psychosocial interventions, as well as outpatient prescription drugs are excluded, which might be contributing to a portion of unmet care needs in this population [9]. Indeed, although to a lesser extent than the United States, financial barriers to mental health services are also reported in Canada [73, 76–78]. Furthermore, and highly concerning, is that under the current Mental Health Act in British Columbia, individuals with presumptive mental illness can be involuntarily admitted to hospital based solely on police officers subjective diagnoses of potential harm to themselves or others (rather than on a professional medical evaluation) [79]. Altogether, these findings raise concern about a potential hidden form of social control, and point to the need to revisit mental health services provision and coverage by Canada’s publicly-funded health system, as well as to explore interventions that take into account the specific needs of patients with mental illnesses in order to improve their access to and quality of mental health services and other support resources, and ultimately reduce health disparities.

Finally, a somewhat unexpected finding of this analysis was that women living with HIV were less likely to report institutional barriers to health care. Historically, women living with HIV have faced multiple barriers to appropriate and quality health care, including HIV related care [80–82]. This is particularly true for key HIV-affected populations, such as women sex workers. Pervasive stigma and discrimination within and outside the health sector, as well as punitive laws targeting sex work, have been identified as important drivers of health care avoidance and sub-optimal HIV treatment outcomes among sex workers [22, 83–85]. However, within the current context in Vancouver, it could be the case that rapid scale up of an intensive government-sponsored HIV prevention and care initiative [86], coupled with the roll-out of women-centred care models [87] might have resulted in better access and engagement in HIV care among women sex workers. This, in turn, might have contributed to improved general care among women living with HIV in our cohort. Yet, in a sub-analysis restricted to sex workers living with HIV, we found that marginalized sub-populations, including sexual/gender minorities, immigrants, and women without a provincial health insurance card were at increased odds of reporting institutional barriers to health services (data not shown), highlighting the need for further research to examine access barriers as they specifically pertain to women sex workers living with HIV. In addition, the evidence documenting the harms associated with criminalization of sex work, including violence by clients and police, increased risk of HIV, as well as food and housing insecurity, should not be overlooked [18, 20, 22, 31, 81]. Nor should we overlook the potential for criminalization of sex work and HIV to continue to promote institutional level barriers to accessing health care through stigma, discrimination, and fear of legal reprisals, which creates serious impediments to a range of primary and secondary prevention initiatives, in addition to HIV treatment efforts. As modelling studies indicate, decriminalization of sex work (as a single intervention) could avert approximately 40% of HIV infections among sex workers and their clients in the next decade in Vancouver [22].

A number of limitations should be considered in the interpretation of the current study. First, due to the criminalized and stigmatized nature of sex work in Canada, the study sample was not randomly selected, and therefore our results might not be generalizable to all sex workers in Vancouver or to other settings with different health systems arrangements or sex work laws. To mitigate this potential selection bias we employed time-location sampling [24, 25], a well-known strategy for achieving representative samples of hard-to-reach and hidden populations. Second, this analysis relied on self-reported data that might have been affected by social desirability, underreporting or recall biases. However, all the interviews were conducted in safe and privacy-enhancing environments by interviewers with extensive experience and strong community rapport (including experiential staff), and we have no reason to believe that there would be differences in the reporting of sensitive data between participants who experienced barriers to health care and those who did not. Third, our primary outcome was based on a self-report measure over a 6-month recall period, which could have resulted in the underestimation of the real prevalence of institutional-level barriers to health care in this sample. Similarly, participants might have experienced other institutional-level barriers (e.g., costs, distance/transportation) that were not included in our questionnaire. Fourth, although this analysis relied on prospective longitudinal data, the study design (e.g., 6-month reference point for both the time updated outcome and explanatory variables) does not permit the establishment of temporal and causal pathways.

In summary, this study found that despite Canada’s universal health system, women sex workers in Vancouver face alarmingly high prevalence of institutional-level barriers to health services, including long wait times, limited hours of operation, and perceived disrespect by health care providers. Further, consistent with the Inverse Care Law [88], some of the most marginalized women and with greatest health care needs in our sample were at increased risk of experiencing these barriers. First and foremost, our findings support global calls to the removal of criminal sanctions against all aspects of sex work to fully fulfill women sex workers health and human rights. In turn, results from this study highlight the need for safe and enabling environments that can promote sex workers’ access to appropriate health services. Globally, there are experiences of proportionate universalism approaches [89], where sex-worker-led, low-threshold service delivery models have been demonstrated to improve access to care, and decrease the numerous health and social inequities faced by this population [27, 52–55]. It is time to take similar action within the Canadian context in terms of our efforts to provide universal access within a globally respected UHC system; too much is at stake to accept the status quo.
